# Sublingual Traumatic Ulceration in a Nine-Year-Old Child: A Case Report

**DOI:** 10.7759/cureus.68107

**Published:** 2024-08-29

**Authors:** Ravikant V Sune

**Affiliations:** 1 Oral Medicine and Radiology, Sharad Pawar Dental College and Hospital, Datta Meghe Institute of Higher Education and Research, Wardha, IND

**Keywords:** permanent mandibular incisors, repetitive trauma, eosinophilic granuloma, chronic traumatic ulcer, riga-fede disease, sublingual traumatic ulceration

## Abstract

Sublingual traumatic ulceration is a rare inflammatory disorder characterized by benign, ulcerative, and granulomatous processes and caused by repetitive trauma from mandibular anterior incisors to the mucosa of the ventral surface of the tongue. Historically known as the Riga-Fede disease, sublingual ulceration is associated with natal and neonatal teeth in newborns and infants. Even cases have been reported of Riga-Fede disease associated with the eruption of mandibular primary incisors. Commonly, the treatment for Riga-Fede disease is the smoothening of incisal edges or the extraction of offending teeth. If this disorder is not diagnosed early and proper treatment is not provided, it may result in dehydration, nutritional deficiency, and deformity of the tongue. Here, we present a case of sublingual traumatic ulceration in a nine-year-old child, which is a rare occurrence. As the associated teeth in our case were permanent mandibular incisors, we opted for useful instructions to avoid repetitive trauma and medicinal treatment to resolve the lesion rather than extraction of teeth. We opted for topical corticosteroids with multivitamin supplements, which proved effective, and we achieved a positive outcome with complete resolution of the lesion following treatment.

## Introduction

Traumatic ulcers on the ventral surface of the tongue are relatively uncommon. This sublingual ulceration is a benign, granulomatous, and ulcerative process that occurs due to repetitive trauma from the teeth to the oral mucosa of the ventral surface of the tongue [1]. Sublingual ulceration, known historically as the Riga-Fede disease, is traumatic ulceration on the ventral surface of the tongue in newborns and infants associated with natal and neonatal teeth [2,3]. Ventral surface lesions are caused by repetitive movements back and forth of the tongue over mandibular anterior incisors [4,5]. Antonio Riga first described this lesion in 1881, and later, histological studies were presented by Francesco Fede in 1890 [1]. Histologically, these lesions are characterized by ulceration and an inflammatory infiltrate consisting of lymphocytes, plasma cells, mast cells, occasional histiocytes, and numerous eosinophils in the granulation tissue stroma [1].

Traumatic ulcers on the ventral surface of the tongue can also occur after the eruption of deciduous mandibular incisors in older infants [6]. Nutritional deficiency and dehydration can occur in infants if the disease is not diagnosed early and proper treatment is not addressed. Treatment for this disease entity should be approached conservatively with the primary aim of removing the source of trauma [6].

Many case reports of sublingual traumatic ulceration have been documented in the literature in newborns and infants associated with natal and neonatal teeth. Additionally, few reports of the same lesion in older infants associated with the eruption of primary mandibular incisors are available. Here, we report a rare case of sublingual traumatic ulceration in a nine-year-old child associated with erupted permanent mandibular incisor teeth.

## Case presentation

**Figure 1:**
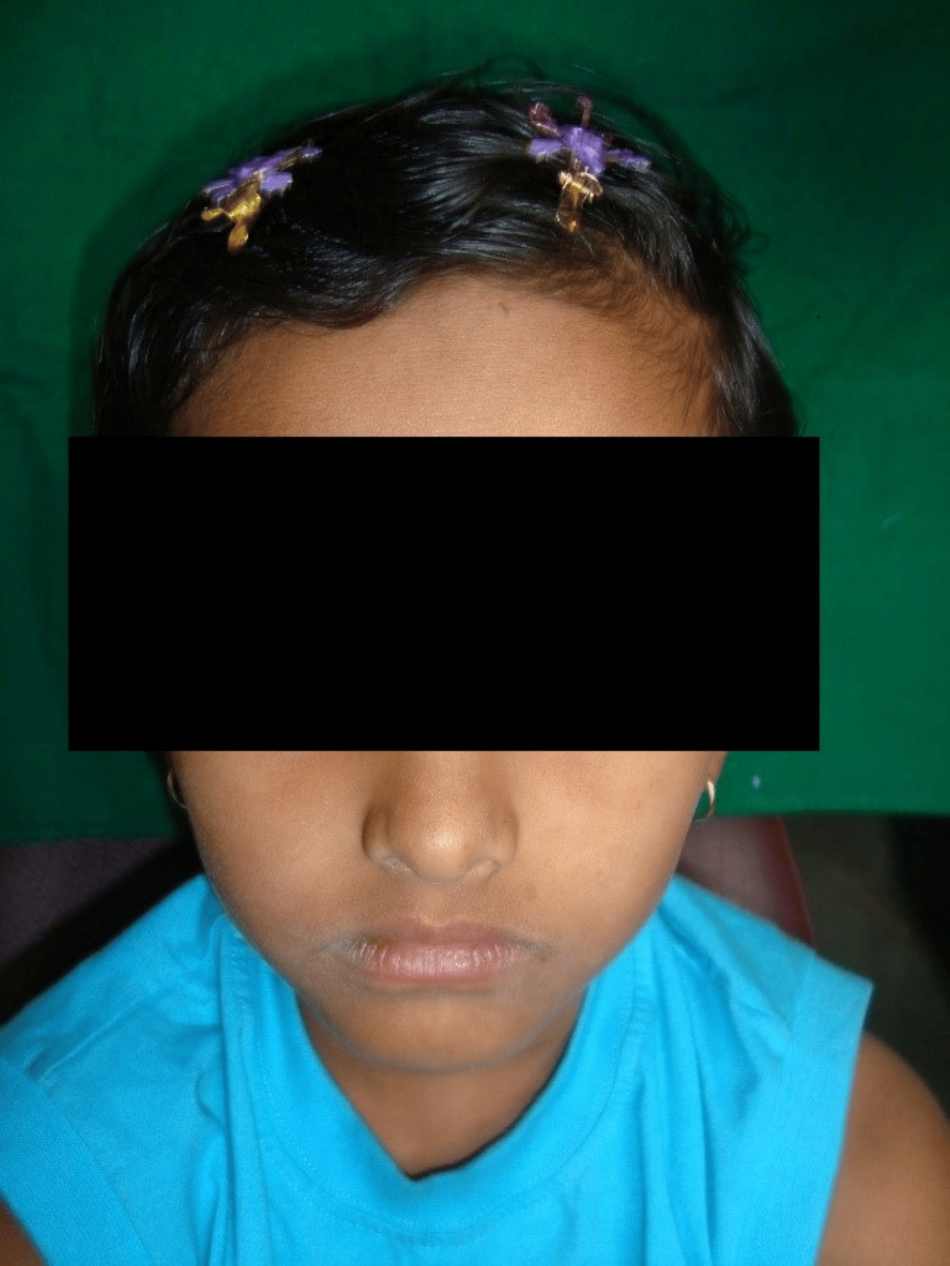
Photograph of the nine-year-old patient

A nine-year-old female patient (Figure 1) reported the chief complaint of an ulcer on the ventral surface of the tongue involving the lingual frenum for two months. The patient gave a history of trauma from pencils, which was confirmed by her parents, as she had a habit of keeping a pen/pencil in her mouth repetitively while studying. Due to unease in the sublingual region, the patient used to rub her tongue against the mandibular incisors. Initially, the ulcer was small and gradually increased in size, which was associated with dull and continuous pain, and the pain used to increase in severity during eating, drinking, and speech. Also, this sublingual ulcer was associated with a burning sensation during food intake. Many times, she used to refuse food due to fear of pain. Then, parents became highly concerned for the health of their child due to frequent refusal of food and the persistence of ulcers. The ulcer was not preceded by fever or a history of systemic illness. The patient was not allergic to any drug, which was confirmed by her parents. The family history of any congenital or developmental abnormalities was also negative.

**Figure 2:**
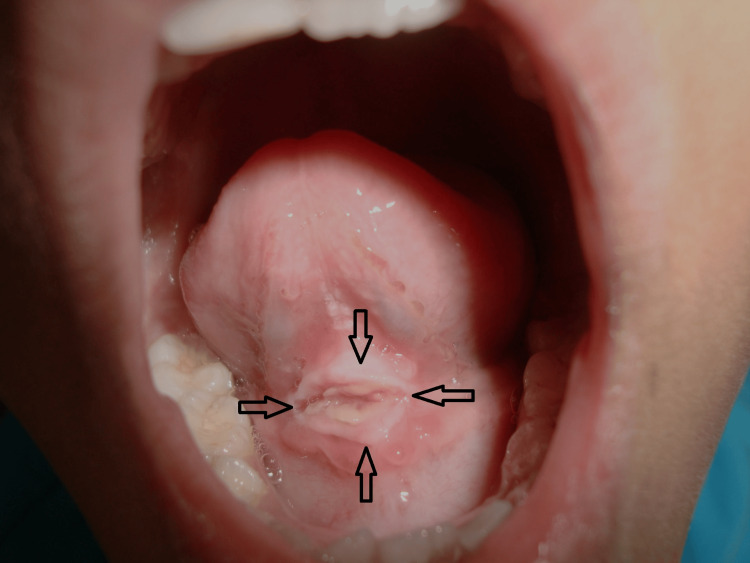
Photograph of the sublingual traumatic ulcer before treatment

On extraoral examination, submandibular lymph nodes were palpable bilaterally, which were one on each side, mobile, nontender, firm, and approximately 0.5 x 0.5 cm in size. All other inspectory and palpatory findings were normal. On intraoral examination, an ulcer was noted on the ventral surface in the midline involving the lingual frenum of size 1.5 x 1 cm with the yellowish-white membrane in the middle surrounded by a white, thick, keratotic border (Figure 2). An area of approximately 1 cm around the border showed erythema. The borders were firm and mildly indurated on palpation, and tenderness was present. There was no bleeding or suppuration from the ulcer. Considering the history of trauma, location, and appearance of the lesion clinically, we gave the diagnosis of sublingual traumatic ulcer.

**Figure 3:**
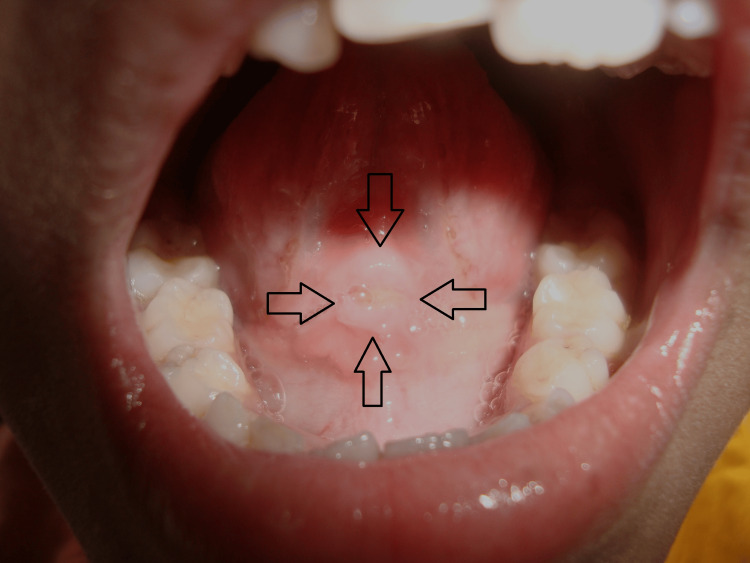
Photograph of the sublingually healed ulcer area after treatment

As the lesion was situated on the ventral surface in the midline involving the lingual frenum, the patient was advised not to stretch the tongue so that it would not rub against the mandibular incisors, with the particular precaution of not holding the pen/pencil in the mouth while studying. For the initial five days, topical application of clotrimazole 1% twice daily and multivitamin oral suspension containing vitamin B complex and vitamin C twice daily were prescribed. Fungal infection is considered the most opportunistic and, in pathologic conditions, appears to penetrate the epithelium. Oral ulceration can easily be caused by secondary infections, such as fungal [7]. Therefore, the antifungal agent was prescribed for five days in the present case. Multivitamin oral syrup was given to improve nutritional intake and rapid healing of the lesion. On a recall visit after five days, the yellowish membrane disappeared completely, but the thick border and erythema at the base and surrounding the lesion were still present. This time, the patient was advised to apply topical triamcinolone acetonide 0.1% twice daily along with chlorhexidine mouth rinse twice daily, with the continuation of multivitamin oral suspension. The patient was recalled after another 10 days. Due to the inflammatory nature of the sublingual traumatic ulcer, topical corticosteroid was prescribed as it is an effective anti-inflammatory agent [8]. On a recall visit after another 10 days, the lesion healed completely. There was no erythema, and the surface appeared epithelized correctly (Figure 3). At this time, the patient was released with the only instruction of not putting pen/pencil intraorally while studying. Both the patient and the parents expressed high satisfaction with the treatment.

## Discussion

A variety of terms have been used in the literature to describe lingual traumatic ulcers, such as Riga-Fede disease, Riga's disease, sublingual growth in infants, sublingual fibro-granuloma, sublingual ulcer, reparative lesion of the tongue, traumatic atrophic glossitis, lingual traumatic ulceration, traumatic granuloma, eosinophilic ulcer, eosinophilic granuloma, and traumatic ulcerative granuloma with stromal eosinophilia [1,9,10]. Ulcers on the ventral surface are caused by repetitive trauma to the tongue over the mandibular anterior incisor teeth [1,4,5]. Sublingual ulceration in newborns and infants is commonly associated with natal and neonatal teeth [2,3]. The initial first description came from Antonio Riga of sublingual traumatic ulcer in 1881, and Fede described the histological findings in 1890. Thereafter, this disease became known as Riga-Fede disease [1]. Histologically, Riga-Fede disease shows granulation tissue surrounding the ulceration with stomal eosinophilia [1]. Sublingual traumatic ulceration can also occur in older infants with eruption of primary mandibular incisors [6]. To the best of our knowledge, no case report of Riga-Fede disease after the eruption of permanent mandibular incisors has been documented in the literature. However, we presented a case of sublingual traumatic ulcer similar to that of Riga-Fede disease in a nine-year-old female patient in which all mandibular permanent incisor teeth have erupted.

Sublingual traumatic ulceration may also occur in children with some neurological or developmental anomaly, including insensitivity to pain, such as familial dysautonomia [11]. It can also occur in other neurologic disorders in which self-mutilation is present, such as Lesch-Nyhan syndrome [12]. Therefore, it becomes necessary to rule out some underlying medical problems in patients with traumatic ulcers on the ventral surface of the tongue. We did not find any neurologic or developmental abnormality in the present case, as the family history was negative for any congenital syndromes or developmental disorders. Riga-Fede disease is a benign ulceration that occurs as a reactive process to repetitive trauma by the teeth to surrounding oral mucosal surfaces. As the tongue is raked over the teeth, ulceration occurs most commonly on the ventral surface in the midline involving the lingual frenum [5]. It has been documented that the presence of natal, neonatal, or even primary teeth, along with infantile intuitive tongue thrusting habits, can result in traumatic sublingual ulceration [3]. However, in the present case, the patient was a nine-year-old child with a sublingual traumatic ulcer associated with erupted permanent mandibular incisors. The typical location of the ulcer on the ventral surface of the tongue in the midline involving the lingual frenum and the history of trauma from pencil as an inciting factor, with the occasional raking of the tongue over the mandibular incisors, suggested it was a sublingual traumatic ulcer similar to that of Riga-Fede's disease.

The lesions to be considered in the differential diagnosis of lingual traumatic ulcer include major aphthous stomatitis, deep fungal infection, bacterial infection such as tuberculosis, primary or secondary syphilis, and squamous cell carcinoma [7,13,14]. Fungal infections can be diagnosed with the help of a microscopic examination of a sample stained with potassium hydroxide or gram stain to detect fungal hyphae. In the culture technique, the sample is placed in a culture medium such as Sabouraud agar to grow the candida species. Diagnosis of syphilis can be achieved through serologic examination for markers or observation of spirochetes in dark field microscopy. A tuberculin purified protein derivative skin test can be done to diagnose tuberculosis. A biopsy can be done to rule out any malignant changes through histopathologic examination [14]. The diagnosis of sublingual traumatic ulcer can be achieved through proper study of case history, clinical examination, and, if required, investigations. After the removal of causative factors, if the ulcer persists for more than two weeks, a biopsy can be done to rule out malignancy [8]. In the present case, the diagnosis of sublingual traumatic ulceration was made based on the history of trauma, the typical sublingual site, and the clinical presentation of the lesion.

Timely detection of sublingual traumatic ulcer is crucial, as failure to diagnose the condition early and provide appropriate treatment can lead to nutritional deficiency, dehydration, and tongue deformity [6,9,10]. Many treatment modalities have been opted for traumatic sublingual ulceration. Excision of the lesion was the earlier treatment due to incorrect diagnosis and failure to identify etiology, but the healing of the lesion occurred spontaneously only after weaning of the infant [15]. Conservative treatment is advocated, such as incisal edge smoothening with an abrasive instrument if there is only mild to moderate irritation to the tongue [16]. Otherwise, similar problems can be solved by bonding a composite layer on the incisal edges of the teeth [17-19]. If the lesion is not resolved within a reasonably short time, extraction of the incisors may become necessary to remove the chronic trauma from the offending teeth [15,20-23].

In the present case, the sublingual traumatic ulceration was associated with permanent mandibular incisors. We avoided grinding the incisal edges of the teeth as it would result in the loss of tooth structure, and even the reduced incisal edge may still contact and traumatize the large denuded area of the ulcer during tongue movement. The child was grown enough to understand and follow the instructions given to her to avoid trauma in the sublingual area. We also did not go for teeth extraction as it would result in permanent loss of the incisor teeth, which would cause aesthetic problems and difficulty in mastication. In such a situation, we presumed medicinal treatment would be more efficient. Therefore, we opted for medicinal treatment and gave proper instructions to the patient.

Oral ulceration can easily be caused by secondary infections, such as fungal. Fungal infection is considered the most opportunistic and, in pathologic conditions, appears to penetrate the epithelium with the help of pseudohyphae [7]. After five days of antifungal treatment, the yellowish membrane disappeared completely. Subsequently, a topical steroid was prescribed as the sublingual chronic traumatic ulcer is an inflammatory lesion. Topical corticosteroid, an anti-inflammatory and immunosuppressive agent, is an important drug in treating immune-mediated ulcerations of the oral cavity [8]. Additionally, chlorhexidine mouth rinse was prescribed to avoid any secondary bacterial or fungal infection. During the recall visit, all the inflammatory components, including erythema, were resolved. Throughout the treatment period, the multivitamin syrup was given to improve nutritional intake and help heal the lesion faster. Instructions were given to the patient, such as not to stretch the tongue so that it will not rub against the mandibular incisor and not to put a pen/pencil intraorally during studying to avoid trauma in the area. The chronic traumatic sublingual ulceration in the present case resolved completely within 15 days after treatment.

## Conclusions

We report a case of sublingual traumatic ulcer in a nine-year-old child, which is rare. In such cases, if not diagnosed early, dehydration and nutritional deficiency may result as the ulcers become painful with time, and the child may become frightened and refuse to eat or drink. Conservative treatments, such as grinding the incisal edges of the teeth and bonding the composite layer on the incisal edge, can be tried. If the lesion does not resolve, extraction of the natal, neonatal, or deciduous anterior incisors is the treatment option. As the child in the present case is nine years old, all the mandibular incisors were permanent, which prohibited us from extracting or grinding the incisal edges of the teeth. Therefore, medicinal treatment was done to resolve the lesion with proper instructions to avoid repetitive trauma to the sublingual area. Topical corticosteroids with multivitamin supplements proved effective medicinal treatment, which we opted for in the present case of a sublingual traumatic ulcer and achieved a positive outcome with complete resolution of the lesion.

## References

[1] Elzay RP (1983). Traumatic ulcerative granuloma with stromal eosinophilia (Riga-Fede's disease and traumatic eosinophilic granuloma). Oral Surg Oral Med Oral Pathol.

[2] Goho C (1996). Neonatal sublingual traumatic ulceration (Riga-Fede disease): reports of cases. ASDC J Dent Child.

[3] Buchanan S, Jenkins CR (1997). Riga-Fedes syndrome: natal or neonatal teeth associated with tongue ulceration. Case report. Aust Dent J.

[4] Baroni A, Capristo C, Rossiello L, Faccenda F, Satriano RA (2006). Lingual traumatic ulceration (Riga-Fede disease). Int J Dermatol.

[5] Choi SC, Park JH, Choi YC, Kim GT (2009). Sublingual traumatic ulceration (a Riga-Fede disease): report of two cases. Dent Traumatol.

[6] Slayton RL (2000). Treatment alternatives for sublingual traumatic ulceration (Riga-Fede disease). Pediatr Dent.

[7] Sella S, Rizal MF (2011). Treatment of lingual traumatic ulcer accompanied with fungal infections. Dental Journal (Majalah Kedokteran Gigi).

[8] Nelonda R, Setiadhi R (2018). Management of chronic traumatic ulcer mimicking oral squamous cell carcinoma on the tongue. Dental Journal (Majalah Kedokteran Gigi).

[9] Ahmet T, Ferruh B, Gürcan A (2003). Lingual traumatic ulceration (Riga-Fede disease). Br J Oral Maxillofac Surg.

[10] Baghdadi ZD (2002). Riga-Fede disease: association with microcephaly. Int J Paediatr Dent.

[11] Rakocz M, Frand M, Brand N (1987). Familial dysautonomia with Riga-Fede's disease: report of case. ASDC J Dent Child.

[12] Fardi K, Topouzelis N, Kotsanos N (2003). Lesch-Nyhan syndrome: a preventive approach to self-mutilation. Int J Paediatr Dent.

[13] Bombeccari GP, Guzzi G, Pallotti F, Porrini M, Giannì AB, Spadari F (2017). Large oral ulcer of tongue related to dental trauma. Stomatologija.

[14] Ceyhan AM, Yildirim M, Basak PY, Akkaya VB, Ayata A (2009). Traumatic lingual ulcer in a child: Riga-Fede disease. Clin Exp Dermatol.

[15] Hegde RJ (2005). Sublingual traumatic ulceration due to neonatal teeth (Riga-Fede disease). J Indian Soc Pedod Prev Dent.

[16] Delbem AC, Faraco Júnior IM, Percinoto C, Delbem AC (1996). Natal teeth: case report. J Clin Pediatr Dent.

[17] Tomizawa M, Yamada Y, Tonouchi K, Watanabe H, Noda T (1989). Treatment of Riga-Fede's disease by resin-coverage of the incisal edges and seven cases of natal and neonatal teeth (Article in Japanese). Shoni Shikagaku Zasshi.

[18] Baghdadi ZD (2001). Riga-Fede disease: report of a case and review. J Clin Pediatr Dent.

[19] Bafna Y, Khandelwal V, Bafna M, Nayak PA (2013). Management of sublingual ulceration in a 12-month-old child. BMJ Case Rep.

[20] Eichenfield LF, Honig PJ, Nelson L (1990). Traumatic granuloma of the tongue (Riga-Fede disease): association with familial dysautonomia. J Pediatr.

[21] Terzioğlu A, Bingül F, Aslan G (2002). Lingual traumatic ulceration (Riga-Fede disease). J Oral Maxillofac Surg.

[22] Jose SC, Abraham KK, Khosla E (2020). Traumatic sublingual ulceration in a newborn. J Res Dent.

[23] Iandolo A, Amato A, Sangiovanni G, Argentino S, Pisano M (2021). Riga-Fede disease: a systematic review and report of two cases. Eur J Paediatr Dent.

